# Disulfide Bonds in the Ectodomain of Anthrax Toxin Receptor 2 Are Required for the Receptor-Bound Protective-Antigen Pore to Function

**DOI:** 10.1371/journal.pone.0010553

**Published:** 2010-05-10

**Authors:** Jianjun Sun, R. John Collier

**Affiliations:** Department of Microbiology and Molecular Genetics, Harvard Medical School, Boston, Massachusetts, United States of America; Columbia University, United States of America

## Abstract

**Background:**

Cell-surface receptors play essential roles in anthrax toxin action by providing the toxin with a high-affinity anchor and self-assembly site on the plasma membrane, mediating the toxin entry into cells through endocytosis, and shifting the pH threshold for prepore-to-pore conversion of anthrax toxin protective antigen (PA) to a more acidic pH, thereby inhibiting premature pore formation. Each of the two known anthrax toxin receptors, ANTXR1 and ANTXR2, has an ectodomain comprised of an N-terminal von Willebrand factor A domain (VWA), which binds PA, and an uncharacterized immunoglobulin-like domain (Ig) that connects VWA to the membrane-spanning domain. Potential roles of the receptor Ig domain in anthrax toxin action have not been investigated heretofore.

**Methodology/Principal Findings:**

We expressed and purified the ANTXR2 ectodomain (R2-VWA-Ig) in *E. coli* and showed that it contains three disulfide bonds: one in R2-VWA and two in R2-Ig. Reduction of the ectodomain inhibited functioning of the pore, as measured by K^+^ release from liposomes or Chinese hamster ovary cells or by PA-mediated translocation of a model substrate across the plasma membrane. However, reduction did not affect binding of the ectodomain to PA or the transition of ectodomain-bound PA prepore to the pore conformation. The inhibitory effect depended specifically on reduction of the disulfides within R2-Ig.

**Conclusions/Significance:**

We conclude that disulfide integrity within R2-Ig is essential for proper functioning of receptor-bound PA pore. This finding provides a novel venue to investigate the mechanism of anthrax toxin action and suggests new strategies for inhibiting toxin action.

## Introduction

A common way for pathogenic bacteria to defend themselves against the host's immune system is to deliver a toxin into the cytoplasm of host cells and disrupt key steps of metabolism. Most intracellularly acting toxins are bipartite entities, in which one part, the B (binding) moiety, binds to a cell surface receptor, hijacks the receptor-mediated endocytosis pathway, and facilitates delivery of the other part, the A (catalytic) moiety, to the cytosol [1]. Study of the roles of receptors in toxin action is of interest in understanding bacterial pathogenesis and in developing novel therapeutics against infection.

Anthrax toxin is a tripartite system, composed of two catalytic moieties, edema factor (EF) and lethal factor (LF), and a receptor-binding/pore-forming moiety, protective antigen (PA). PA (83 kDa) binds to cell-surface receptors and is cleaved by furin or a furin-like protease to generate an active, 63-kDa form (PA_63_) [2]. PA_63_ oligomerizes into a heptameric or octameric [3], receptor- bound prepore, which contains high-affinity binding sites for EF and LF[4]. The toxin-receptor complexes are internalized by receptor-mediated endocytosis, and the prepore moiety undergoes an acidic pH-dependent conformational rearrangement within the endosome to form a cation-selective, transmembrane pore [5]. The PA pore mediates translocation of EF and LF across the endosomal membrane into the cytosol, where, EF, an 89-kDa calmodulin-dependent adenylate cyclase, elevates levels of cAMP [6], and LF, a 90-kDa zinc protease, inactivates mitogen-activated proteins kinase kinases [7].

**Figure 1:**
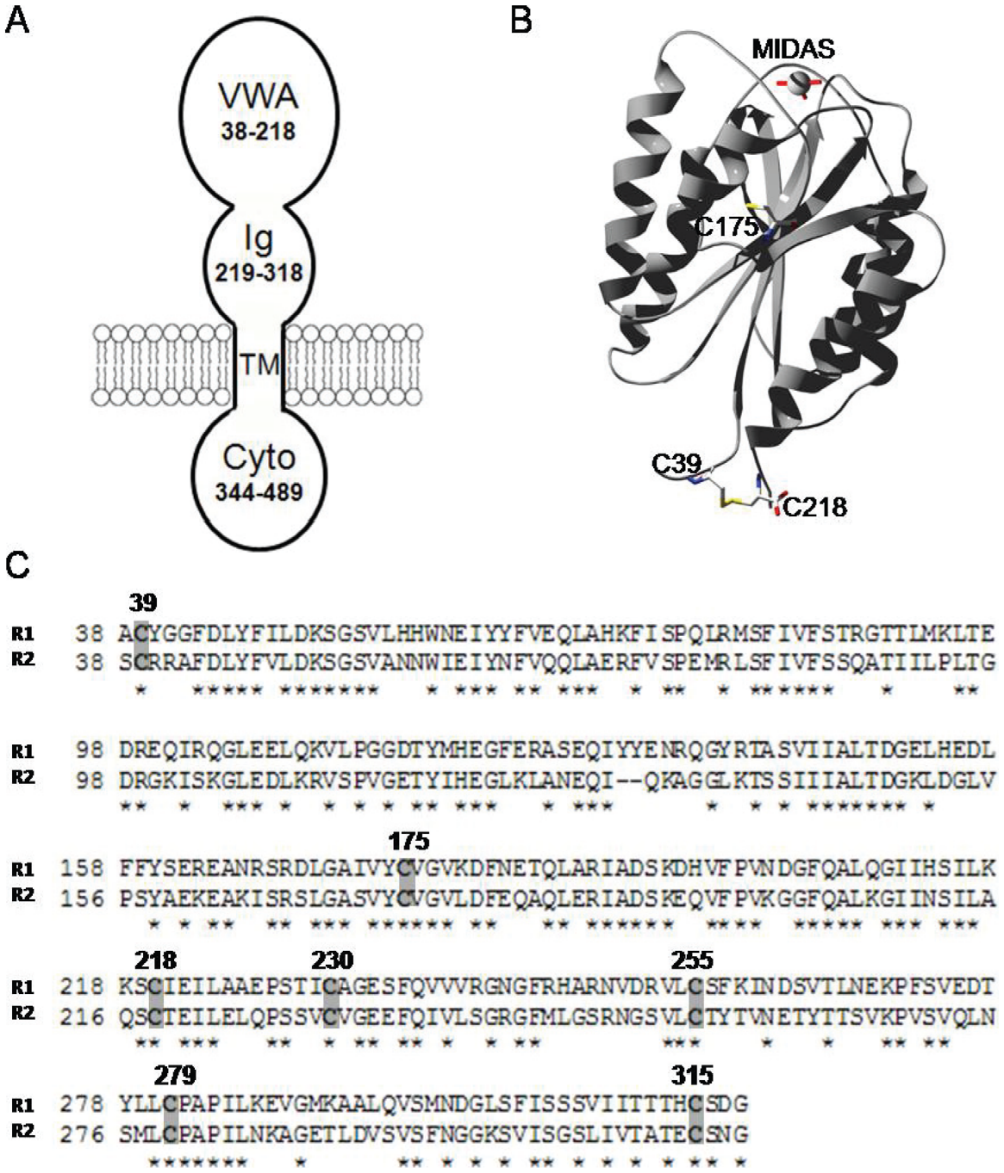
ANTXR2 ectodomain is composed of R2-VWA and R2-Ig. **A**. Schematic of ANTXR2. ANTXR2 is composed of R2-VWA (VWA, 38–218) and R2-Ig (Ig, 219–318), a single-pass transmembrane domain (TM, 319–343) and a cytoplasmic domain (Cyto, 344–489). **B**. The crystal structure of R2-VWA domain (1SHU) as displayed in Swiss-PDB-Viewer. Residues Cys175, the Cys39–Cys218 disulfide bond, and Mn^2+^ ion of MIDAS are shown and labeled. **C**. Sequence alignment of the ectodomains of ANTXR1 (R1) and ANTXR2 (R2). The seven conserved Cys residues are highlighted and numbered in R2. Identical residues are labeled with asterisk (*).

Two cellular receptors for PA have been identified: ANTXR1 [8] and ANTXR2 [9]. Recently, it has been shown that the lethality of anthrax toxin for mice is primarily mediated by ANTXR2 and that ANTXR1 plays only a minor role [10]. Both receptors are widely expressed type-I transmembrane proteins, which exhibit a high degree of similarity; each comprises an extracellular domain (ectodomain), a single-pass transmembrane domain, and a cytoplasmic domain (**Figure 1A**). PA binds to a von Willebrand Factor type A (VWA) domain located at the N terminus of the ectodomain. While the physiological functions of the receptors have not been fully elucidated, they bind to extracellular matrix components and are associated with angiogenesis [11]–[13]. Mutations in the ANTXR2 gene are linked to two human autosomal recessive diseases, juvenile hyaline fibromatosis and infantile systemic hyalinosis [14]–[17].

A conserved metal ion-dependent adhesion site (MIDAS) within R2-VWA, the VWA domain (residues 38–218) of ANTXR2, binds PA with high affinity (*K_d_*∼170 pM when liganded by Mg^2+^) (**Figure 1B**) [18]–[20]. Binding to R2-VWA involves not only domain 4 (receptor binding domain) of PA, but also domain 2 (pore-forming domain) [18]. The 2β2–2β3 loops (residues 303–322) of the domains 2 of heptameric PA prepores move to the base of the structure during the low pH-induced conformational rearrangement and form a 14-strand transmembrane β-barrel [21]. Binding of R2-VWA to the PA prepore shifts the pH threshold of prepore-to-pore conversion to a more acidic pH range [20], [22]. Whole-cell voltage-clamp measurements of PA pore current have shown that ANTXR2 mediates PA pore formation on the plasma membrane of cultured cells when they are exposed to acidic conditions [23].

While R2-VWA has been well characterized, the structure and function of the domain (residues 219–318) downstream of R2-VWA within the extracellular portion of ANTXR2 have not yet been addressed. A reverse position-specific BLAST (RPS-BLAST) search against the conserved domain database shows that this domain is a member of the immunoglobulin (Ig) superfamily (pfam05587, here termed R2-Ig) (**Figure 1A**). Within the ANTXR2 ectodomain, there are seven Cys residues, with three on R2-VWA and four on R2-Ig (**Figure 1C**). These residues are highly conserved among the homologues of anthrax toxin receptors. Our current study indicates that the integrity of the disulfide bonds in R2-Ig is essential for proper functioning of the receptor-bound pore, and presumably for receptor-mediated anthrax toxin action.

## Materials and Methods

### Cell Culture and Media

An anthrax toxin receptor-deficient CHO-R1.1 cell line that stably expresses ANTXR2-GFP (termed CHO-ANTXR2) was a gift from John A. T. Young [8], [9]. The cells were grown in Ham's F-12 medium supplemented with 10% calf serum, 2 mM glutamine, 500 units/ml penicillin, 500 units/ml streptomycin sulfate, and 100 units/ml G418, under a humidified atmosphere with 5% CO_2_.

### Plasmid Construction and Protein Purification

The cDNA encoding residues 38–318 of ANTXR2 was amplified by PCR and inserted into a pMAL-p2x vector (NEB) at Xho I and Hind III sites with a His6-tag at the C-terminus flanked by a thrombin cleavage site. The construct expresses a fusion protein, MBP-ANTXR2(38–318)-His_6_. The fusion protein was co-expressed with a bacterial disulfide isomerase, DsbC (a kind gift from Jon Beckwith), in the periplasm of bacterial strain BL21(DE3). Bacterial cells were inoculated in a 10 L-fermentor in a rich medium at 37°C until OD_600_ reached ∼10. The temperature was shifted to 16°C and expression of the fusion protein and DsbC were induced with 0.1 mM IPTG and 0.2% arabinose, respectively, for overnight. The bacterial cells were harvested, and the periplasmic fraction was prepared by osmotic shock as previously described [20]. The fraction was passed through a Ni-column and eluted with an imidazole gradient. The eluted protein was concentrated, and the soluble monomeric form of the fusion protein was further purified by gel filtration on a Superdex-200 column. Subsequently, the MBP- and His_6_- tags were removed by incubating the protein with Factor Xα and thrombin, respectively. The recombinant PA and R2-VWA were expressed and purified as described [20].

### Estimation of free–SH group on R2-VWA-Ig with Ellman's reagent

Ellman's reagent (5,5′dithiobis(2-nitrobenzoic acid); DTNB; 500 µM) was incubated with 10 µM of R2-VWA-Ig (WT) or R2-VWA-Ig (C175A) in 6 M guanidinium chloride and 0.1 M TrisHCl (pH 8.0). The absorbance at 412 nm was recorded in a spectrophotometer. Similarly, L-Cysteine at various concentrations (0, 2.5, 5, 10, 20, 40, 80, 160 µM) was incubated with 500 µM of Ellman's reagent in 6 M guanidinium chloride and 0.1 M TrisHCl (pH 8.0), and the absorbance at 412 nm was recorded. These data were used to plot a standard curve. The concentration of free–SH group ([-SH]) in the protein samples was calculated based on the standard curve, and the number of–SH group per molecule was calculated as [-SH]/[protein].

### Gel Shift Assay for PA-Receptor Binding

PA_83_, R2-VWA, and R2-VWA-Ig were mixed (receptor/PA ratio 2∶1), as indicated, in 20 mM Tris-HCl (pH 8.5), 150 mM NaCl, 1 mM MgCl_2_, with or without 10 mM TCEP, at room temperature for 30 min. The samples were loaded onto 4–20% acrylamide, Tris-glycine gels (Invitrogen). The running buffer was 25 mM Tris-base, 192 mM glycine. The gels were stained with Coomassie blue. Note: treating with either DTT or TCEP before or after receptor binding to PA gave the same results. Thus, neither the choice of the reducing agent nor the timing of addition of the reducing agent had any effect on the results.

### SDS-Resistant PA_63_ Oligomer

To assay PA prepore to pore transition in solution, PA_63_ heptameric prepore, R2-VWA, and R2-VWA-Ig were mixed (receptor/PA ratio 2∶1), as indicated, in 20 mM Tris-HCl (pH 8.5), 150 mM NaCl and 1 mM MgCl_2_, with or without 10 mM TCEP, at room temperature for 30 min. The solution was acidified by the addition of 1/10 volume of 1 M sodium acetate (pH 5.0) and incubated for 10 min. Samples were exposed to 1.25% SDS for 20 min and electrophoresed on SDS-PAGE, and the bands were visualized after Coomassie staining. To assay PA prepore to pore transition on the plasma membrane, CHO-ANTXR2 cells were incubated with PA_63_ prepore (10 µg/ml), with or without 10 mM DTT or TCEP, for 1 h at 4°C. The cells were washed with cold PBS and incubated in 150 mM NaCl, 20 mM Mes, 5 mM gluconic acid (pH 5.0) for 10 min at 4°C. The cells were then harvested and exposed to 1.25% SDS at 100°C for 10 min. The samples were subjected to SDS-PAGE followed by Western blotting with a goat anti-PA antibody (List Biolab), mouse anti-goat horseradish peroxidase (Santa Cruz), and SuperSignal Western detection reagent (Pierce).

### Liposome Preparation

Liposomes were prepared as previously described [24]. Briefly, DOPC in chloroform (Avanti Polar Lipids) was dried under N_2_ gas to form a lipid film, followed by vacuum for 3 h to remove residual solvent. The dried lipid film was rehydrated with buffer to form multilamellar vesicles and subjected to six freeze-thaw cycles and extrusion through a 200-nm pore size polycarbonate filter (Nucleopore) in a mini extruder (Avanti Polar Lipids). The protocol yielded large unilamellar vesicles with an average diameter of 150–200 nm.

### K^+^ Release From Liposomes and Cells

Liposome containing 150 mM KCl, 10 mM Hepes (pH 7.4) were transferred into 150 mM NaCl, 20 mM Tris-HCl (pH 8.5) by buffer exchange. The liposome was added to K^+^ release buffer (50 mM sodium acetate, 150 mM NaCl, pH 5.0) and after 1 min, PA prepore (3 nM) complexed with either R2-VWA or R2-VWA-Ig (40 nM) was added. The receptor proteins were treated with 10 mM DTT or TCEP before or after incubating with PA prepore. The solution was stirred continuously with a magnetic stirrer, and K^+^ release was monitored with a K^+^-selective electrode (Orion Research).

### PA-Mediated LF_N_ Translocation Across the Plasma Membrane

As described [25], [26], CHO-ANTXR2 cells were incubated with PA (10 µg/ml) for 2 h at 4°C, with or without the reducing agents. Cells were then washed with cold PBS to remove unbound protein, and ^35^S-LF_N_, produced from TNT coupled reticulocyte lysate system (Promega), was added. The cells were then incubated for 2 h at 4°C, with or without the reducing agents. The unbound ^35^S-LF_N_ was removed by washing, and ^35^S-LF_N_ translocation was triggered by acidification of the cells with pH 5.0 buffer [150 mM NaCl, 20 mM Mes, 5 mM gluconic acid (pH 5.0)] at 37°C for 2 mins. Pronase (2 mg/ml) was added to remove LF_N_ remaining on the cell surface after translocation. The cells were harvested, and the lysates were applied to SDS-PAGE followed by autoradiography.

## Results

### Purification of recombinant ANTXR2 ectodomain, R2-VWA-Ig, from *E. coli*

**Figure 2:**
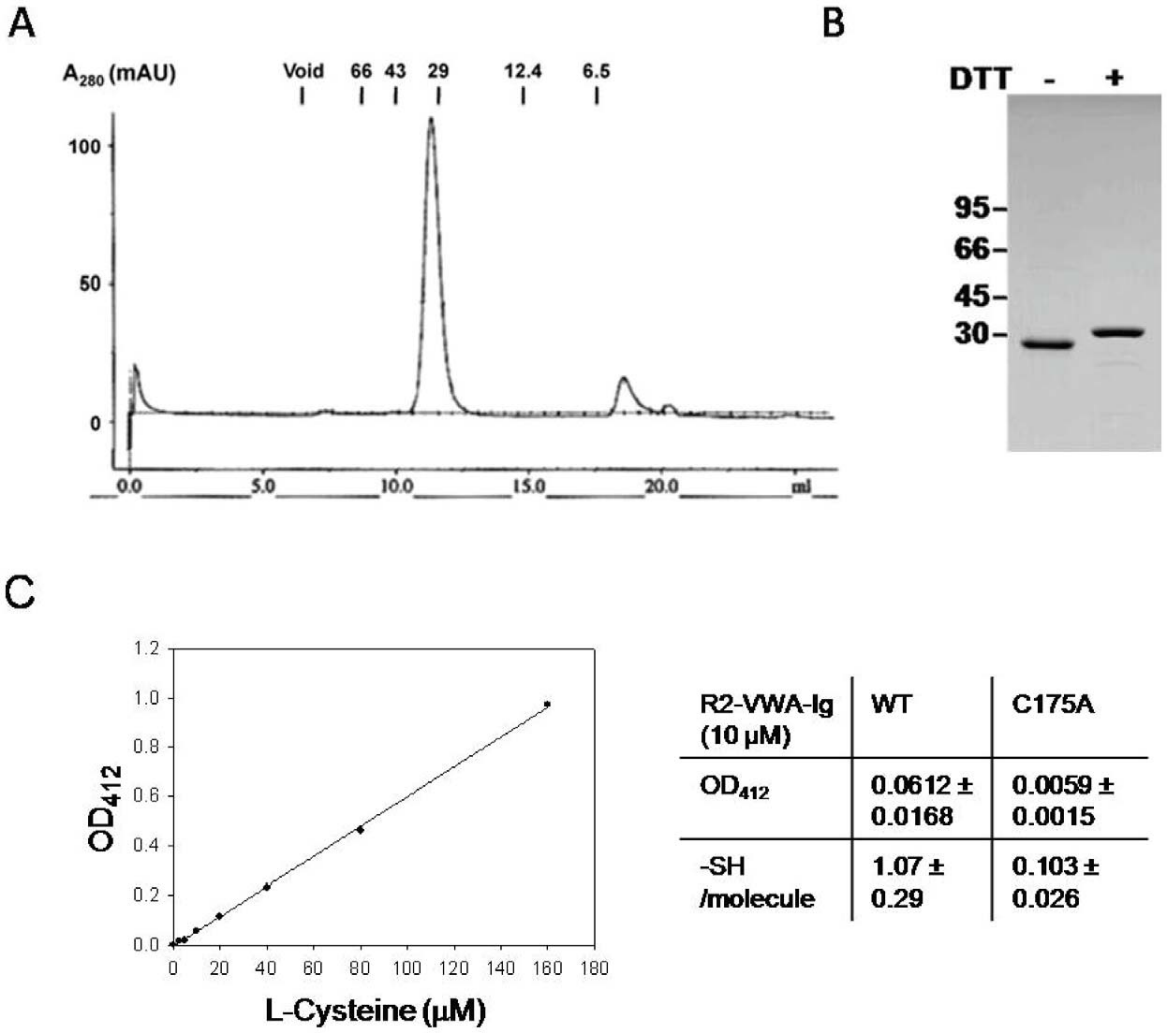
Purified ANTXR2 ectodomain, R2-VWA-Ig, is a soluble monomeric protein containing three disulfide bonds. **A**. Gel filtration of the purified R2-VWA-Ig. R2-VWA-Ig was expressed and purified as a fusion protein with an N-terminal MBP tag and a C-terminal His_6_-tag. After removal of the MBP- and His_6_- tags, R2-VWA-Ig ran as a monomer (∼30 kDal) in a Superdex-75 column in 20 mM TrisHCl (pH 8.0) and 150 mM NaCl. **B**. SDS-PAGE of the purified R2-VWA-Ig. The purified R2-VWA-Ig was treated with or without 10 mM DTT in SDS sample buffer, and run on SDS-PAGE. **C**. left panel, cysteine standard curve; at OD_412_ was plotted after L-cysteine at various concentrations was incubated with 500 µM DTNB in 6 M guanidium HCl and 0.1 M TrisHCl, pH 8.0; right panel, 10 µM of R2-VWA-Ig (WT) or R2-VWA-Ig (C175A) was incubated with 500 µM DTNB in 6 M guanidium HCl and 0.1 M TrisHCl, pH 8.0. OD_412_ was recorded and –SH/molecule was calculated based on the cysteine standard curve and protein concentration.

To express soluble, functional ectodomain of ANTXR2 in *E. coli* and facilitate its purification, we engineered a construct that expressed a MBP-ANTXR2(38–318)-His_6_ fusion protein in the bacterial periplasm, where disulfide bond formation is favored. Co-expression of this construct with a bacterial disulfide isomerase, DsbC, at low temperature increased the yield of soluble protein. After affinity purification through a Ni-column, the MBP- and His_6_- tags were removed with Factor Xα and thrombin, respectively. The purified protein ran at ∼30 kDa on a size exclusion column (**Figure 2A**) and on SDS-PAGE (**Figure 2B**), consistent with the calculated molecular weight of a monomer. The amino acid sequence of the purified protein was confirmed by MALDI-TOF analysis, with >93% peptide recovery (data not shown). When DTT (final concentration, 10 mM) was added to the sample, the protein migrated more slowly in SDS-PAGE, consistent with the reduction of intra-molecular disulfide bonds (**Figure 2B**). The ANTXR2 ectodomain has seven Cys residues in total with three on R2-VWA and four on R2-Ig (**Figure 1C**). In the crystal structure of R2-VWA, Cys39 and Cys218 are linked by a disulfide bond, while Cys175 is unpaired and buried within the structure (**Figure 1B**) [18], [19]. Subsequently, an assay with Ellman's reagent (DTNB) was performed to measure the free–SH content of R2-VWA-Ig against a series of Cys amino acid standards (**Figure 2C**). The results suggested that wild type R2-VWA-Ig contains one reactive–SH group per molecule, while the mutant R2-VWA-Ig (C175A) has none. Therefore, the four Cys residues within the R2-Ig domain (C230, C255, C279, and C315) presumably form two disulfide bonds. Replacing the Cys residues in R2-Ig with Ala residues resulted in expression of the ectodomain in the form of inclusion bodies in *E. coli* (data not shown).

### Reduction of the ectodomain does not affect its ability to bind PA

**Figure 3:**
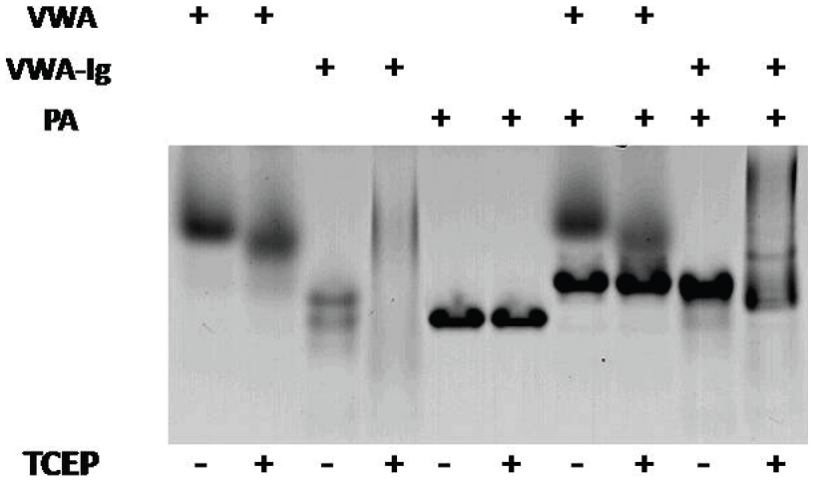
Disulfide reduction of R2-VWA-Ig did not affect receptor-PA binding. PA_83_, R2-VWA, and R2-VWA-Ig were mixed, as indicated in 20 mM Tris-HCl (pH 8.5), 150 mM NaCl, 1 mM MgCl_2_, in the presence or absence of 10 mM TCEP. The samples were subjected to native gel electrophoresis followed by Coomassie blue staining.

R2-VWA and R2-VWA-Ig were tested in a gel shift assay to assess their ability to bind to PA under oxidizing and reducing conditions. Without reducing agent, both R2-VWA and R2-VWA-Ig bound PA, as evidenced by a shift in PA migration in native gel electrophoresis (**Figure 3**). Treatment with reducing agent did not affect binding of R2-VWA or R2-VWA-Ig to PA, even when the treatment was initiated before incubation with PA. TCEP-treated R2-VWA-Ig ran as a smear, suggesting formation of heterogeneous oligomers of R2-VWA-Ig during electrophoresis, and the complexes with PA showed a similar, smeared pattern. The disulfide bond Cys39-Cys218 in R2-VWA had been shown earlier not to be required for PA binding, given that a truncated variant of R2-VWA (residues 40–217, without the disulfide bond) had nearly identical crystal structure and affinity for PA, as wild type R2-VWA (residues 38–218, with the disulfide bond) [18], [20]. We conclude that the integrity of disulfide bonds in the R2-VWA-Ig is not required for receptor-PA binding.

Without reducing agents the recombinant R2-VWA-Ig protein ran as a single monodisperse peak in gel filtration and migrated as a single band in SDS-PAGE (**Figure 2A and B**). However, the protein appeared as a doublet in native gel electrophoresis (**Figure 3**). The basis of this molecular heterogeneity in native gel electrophoresis is not clear and may be related to disulfide isomerization in R2-Ig.

### Reduction does not affect formation of SDS-resistant PA_63_ oligomers

**Figure 4:**
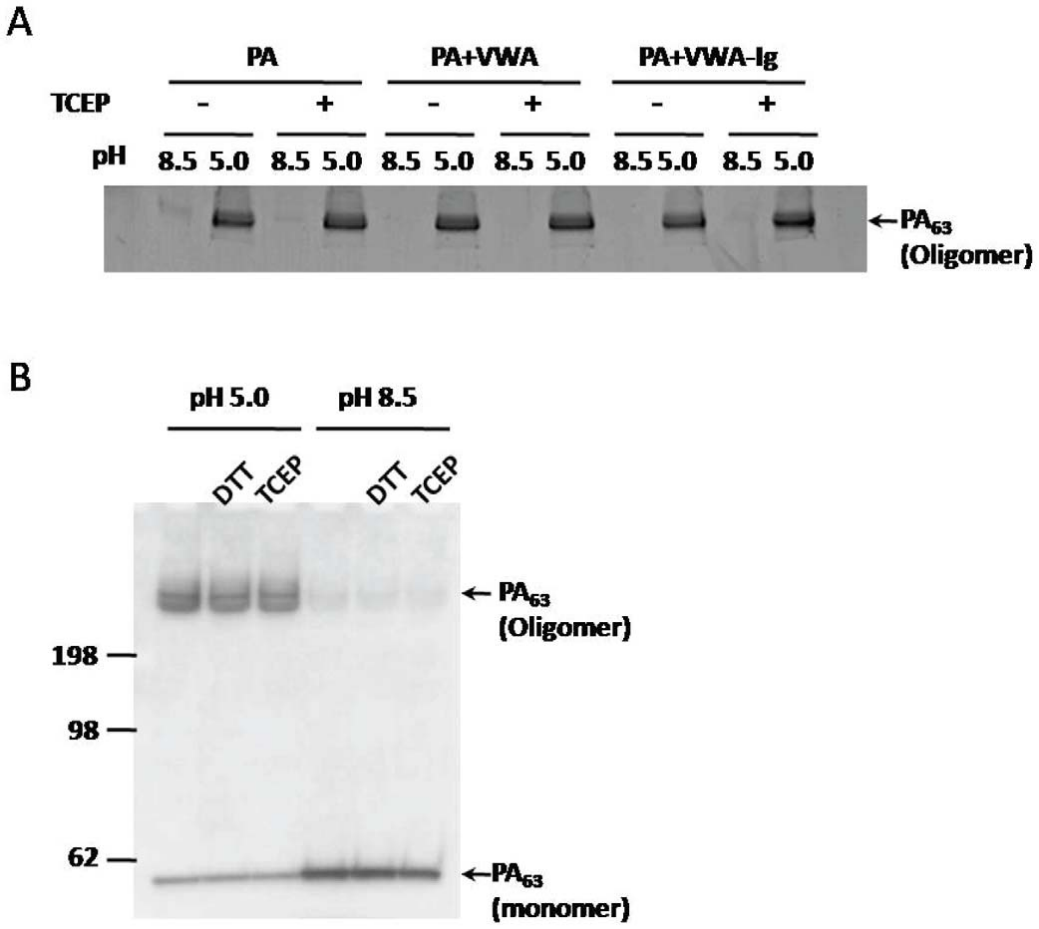
Disulfide reduction did not affect formation of SDS-resistant PA_63_ oligomers at low pH. **A**. PA prepore, R2-VWA, and R2-VWA-Ig were mixed as indicated (receptor/PA ratio is 2/1) in 20 mM Tris-HCl (pH 8.5), 150 mM NaCl, 1 mM MgCl_2_, in the presence or absence of 10 mM TCEP. Acidification was initiated by addition of 1/10 volume 1 M sodium acetate (pH 5.0). The samples were subjected to SDS-PAGE and stained with Coomassie blue. **B**. PA prepore was incubated at 4°C with CHO-ANTXR2 cells in the presence or absence of reducing agents (10 mM DTT or TCEP as indicated). The cells were washed to remove unbound PA, and acidification was initiated by addition of a pH 5.0 buffer into the cell cultures. After 10 min at 4°C the cells were harvested, and lysates were applied to SDS-PAGE, followed by western blotting with anti-PA antibody.

**Figure 5:**
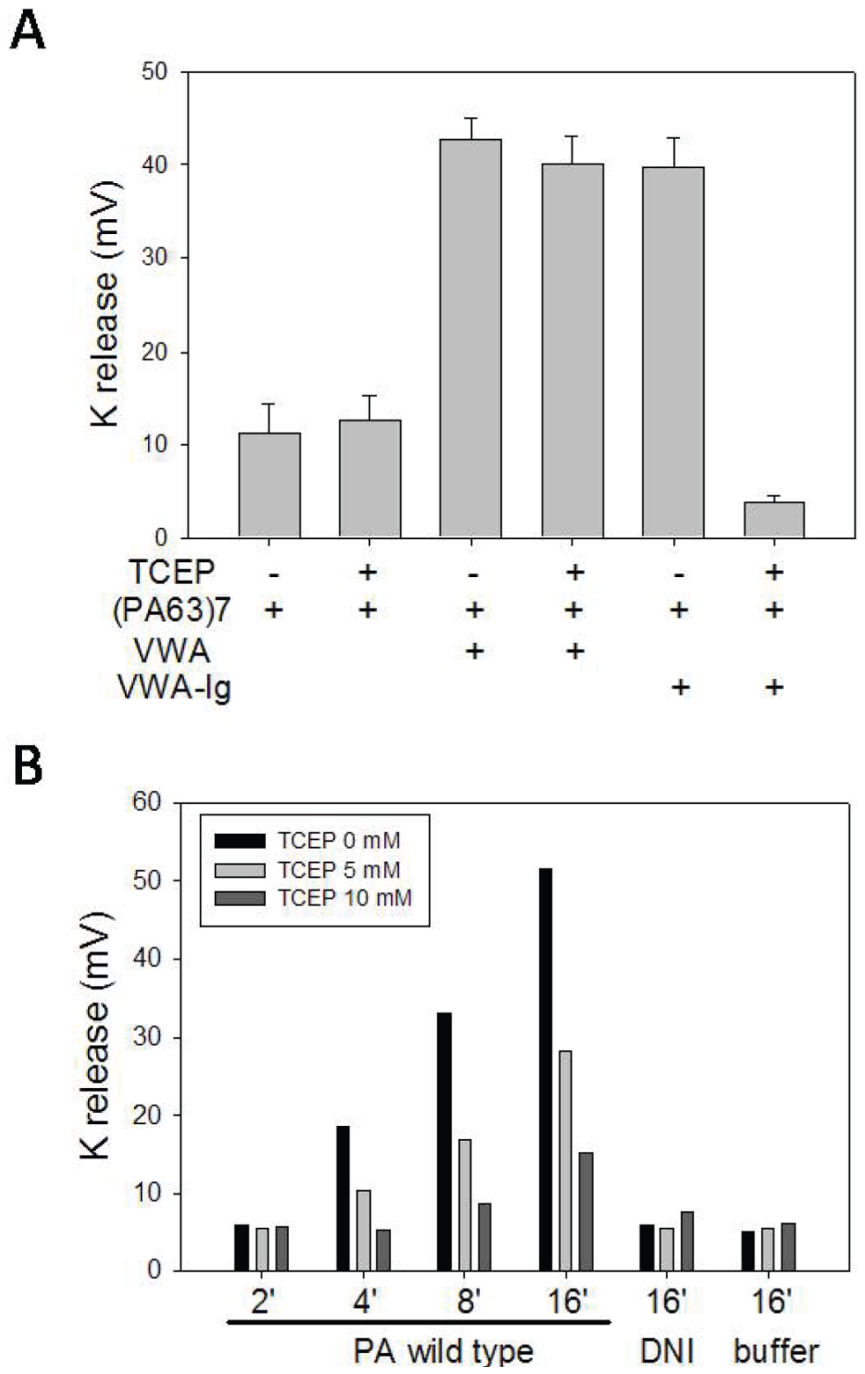
Disulfide reduction of R2-VWA-Ig inhibited release of K^+^. **A**. PA prepore, R2-VWA, and R2-VWA-Ig were incubated as indicated (receptor/PA ratio = 2/1) in 20 mM Tris-HCl (pH 8.5), 150 mM NaCl, 1 mM MgCl_2_, in the presence or absence of 10 mM TCEP. Release of K^+^ ions from KCl-charged liposomes was monitored with a K^+^-selective probe in the solution. **B**. PA prepore (10 µg/ml) was incubated at 4°C with CHO-ANTXR2 cells, with various concentrations of TCEP as indicated. The cells were washed with 20 mM Tris-HCl (pH 8.0) and 150 mM NaCl to remove unbound PA, and acidification was initiated by addition of a pH 5.0 buffer into the cell cultures at 4°C for various times. The supernatants were collected and the content of K^+^ ion was measured by the K^+^-selective probe and read with a pH meter as mV. DNI, a PA dominant negative mutant that is defective in pore formation, served as negative control.

Coincident with the conformational transition of heptameric prepore to pore, triggered by acidic pH, the soluble oligomeric PA_63_ prepore is transformed into an SDS-resistant form [22], [27], [28], and this transformation may be used to monitor the transition. While no SDS-resistant PA_63_ oligomer was observed at pH 8.5, prepore alone, prepore complexed with R2-VWA, and prepore complexed with R2-VWA-Ig were converted to SDS-resistant PA_63_ oligomers at pH 5.0 in the presence or absence of reducing agent (TCEP), as determined by SDS-PAGE (**Figure 4A**). In addition, we tested whether reduction of disulfides of ANTXR2 affected the conformational transition of prepore to pore on the plasma membrane. We used an anthrax toxin receptor-deficient CHO cell line that stably expressed ANTXR2-EGFP (CHO-ANTXR2) [9]. PA prepore was incubated with the cells at 4°C to minimize receptor-mediated endocytosis and maintain PA prepore at the cell surface. Addition of acidic buffer (pH 5.0) to the medium triggered the prepore to pore transition, as evidenced by formation of SDS-resistant PA_63_ oligomers (**Figure 4B**), and this transition was unaffected by DTT or TCEP. Data in Figure 5 imply that disulfides in ANTXR2-EGFP were in fact reduced under the conditions employed. Note that the reducing agents had no effect on PA binding to the ANTXR2 on the cell surface (**Figure 4B**).

### Disulfide reduction of R2-VWA-Ig inhibits the release of K^+^ through the PA pore

As a test of pore function we measured release of K^+^ through PA pores formed in liposomal membranes with PA prepore bound to R2-VWA-Ig (**Figure 5A**). In an earlier communication we showed that binding of R2-VWA to the prepore inhibited aggregation of the pore complexes in solution at low pH, and thereby promoted partitioning of PA into liposomal membranes and formation of ion-permeable pores [24]. Consistent with the previous result, prepore complexed with R2-VWA (PA/R2-VWA) induced greater release of K^+^ than prepore alone. In the absence of reducing agent, prepore complexed with R2-VWA-Ig (PA/R2-VWA-Ig) released K^+^ to a similar level as prepore complexed with R2-VWA. However, reducing agent significantly inhibited K^+^ release by PA/R2-VWA-Ig, but not by PA/R2-VWA. Note that in the presence of TCEP, K^+^ release by PA/R2-VWA-Ig was even lower than that by prepore alone. This finding indicates that reduction of disulfide bonds in the R2-Ig domain, but not that in R2-VWA, inhibits release of K^+^ through PA pore.

We also tested the effect of the reducing agents on K^+^ release through PA pores formed on the plasma membrane of CHO-ANTXR2 cells (**Figure 5B**). After prepore was bound to ANTXR2 on the cell surface, acidification induced K^+^ release from the cells in a time-dependent manner. DNI, a double PA mutant (K397D, D425K) known to be strongly defective in pore formation [29], [30], failed to induce K^+^ release under the same conditions. K^+^ release by cell-bound wild-type prepore was inhibited by TCEP in a dose- and concentration-dependent manner, even if the cells were treated with the reducing agent after PA binding. Thus, disulfide reduction of the R2-Ig domain inhibited the release of K^+^ through PA pore both in liposomes and in a cellular system.

### Disulfide reduction inhibits PA-mediated LF_N_ translocation across the plasma membrane

**Figure 6:**
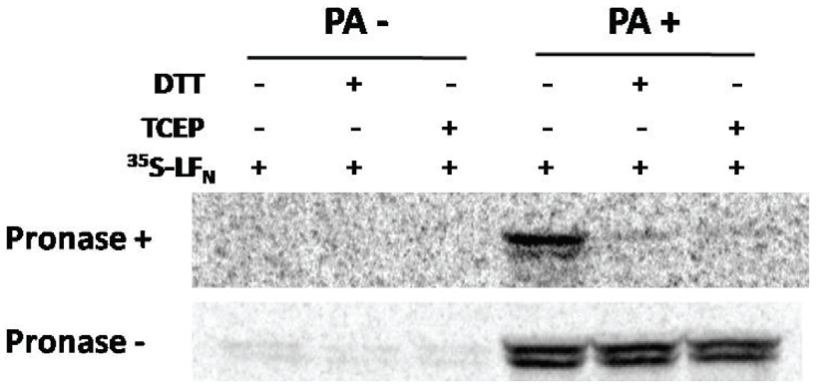
Reducing agents inhibited PA-mediated LF_N_ translocation across the plasma membrane. PA prepore was incubated with CHO-ANTXR2 cells at 4°C followed by the addition of ^35^S-LF_N_ to the cells. The cells were treated with 10 mM DTT or TCEP, as indicated. Translocation was triggered by addition of a pH 5.0 buffer to the cells. The cells were either directly lysed (pronase−) or treated with pronase (pronase+) followed by lysis. ^35^S-LF_N_ was detected by autoradiography.

The effects of DTT and TCEP on K^+^ release through PA pore prompted us to test effects of receptor disulfide reduction on anthrax toxin-mediated cytotoxicity. In a previously developed assay, PA mediates translocation of LF_N_-DTA, a fusion protein composed of LF PA-binding domain and diphtheria toxin A domain, across the endosomal membranes to the cytosol, where LF_N_-DTA inhibits protein synthesis ([^3^H]-leucine incorporation) [31], [32]. However, this assay was hampered by the fact that the reducing agents alone (TCEP and DTT) significantly inhibited protein synthesis under the assay conditions used. Thus, as an alternative we tested whether the reducing agents inhibited PA pore-mediated LF_N_ translocation across the plasma membrane. We used a pronase protection assay [25], [26]. Briefly,^ 35^S-LF_N_ is bound to PA prepore on the cell surface, and upon exposure of the cells to low pH, the labeled protein translocates across the plasma membrane and becomes inaccessible to added pronase. As shown in **Figure 6**, both reducing agents strongly inhibited PA-mediated LF_N_ translocation. Note that, without pronase, equivalent amounts of LF_N_ were detected in the cell lysate with or without the reducing agents, indicating that LF_N_ binding to PA-receptor complexes on the cell surface was not compromised by reduction of the receptor disulfides.

## Discussion

PA binds to cell surface receptors, hijacks receptor-mediated endocytosis pathways, and forms a protein conductive pore on the endosomal membrane. ANTXR2 provides the toxin with a high-affinity anchor and self-assembly site on the plasma membrane and guides its entry into cells; and further the receptor functions as a molecular clamp that shifts the pH threshold of prepore-to-pore conversion to a more acidic pH, thereby preventing premature pore formation before the toxin reaches a suitable compartment. In the present study we have shown that reduction of the ectodomain of the receptor blocks proper functioning of the PA pore. This finding suggests that anthrax toxin action is sensitive to redox conditions and raises the possibility that toxin action may be modulated by reductants to which it is exposed during endocytosis and intracellular trafficking.

A conserved domain search showed that the ectodomain of ANTXR2 contains two distinct domains, R2-VWA and R2-Ig. Based on the crystal structure of R2-VWA and measurements with Ellman's reagent, we predict that R2-VWA-Ig contains three disulfide bonds—one in R2-VWA and two in R2-Ig—plus a buried free Cys, Cys175, in R2-VWA. We found in the absence of reducing agents that R2-VWA-Ig, like isolated R2-VWA, bound PA (**Figure 3**) and enhanced the release of K^+^ from liposomes (**Figure 5A**). R2-VWA-Ig retained the ability to bind to PA in the presence of reducing agents (**Figure 3** **)**, consistent with the fact that the disulfide bond C39-C218 in R2-VWA is not required for PA binding [19], [20]. These data support our predicted distribution of disulfides in the two domains and suggest that the VWA domain behaves the same within the context of R2-VWA-Ig as it does as an isolated domain.

We found a strong inhibitory effect of reduction of R2-VWA-Ig on PA-mediated release of K^+^ from liposomes, whereas PA binding and conversion of bound prepore to the SDS-resistant state were insensitive to this modification. Furthermore, treatment of CHO-ANTXR2 cells with reducing agents blocked PA-dependent K^+^ release and PA-dependent translocation of LF_N_ across the plasma membrane. Reduction did not compromise LF_N_ binding to PA-receptor complexes on the cell surface, suggesting that the inhibition of PA-dependent K^+^ release and translocation resulted directly from reduction of disulfides in cellular ANTXR2 ectodomain.

How might reduction of disulfides in R2-VWA-Ig, and specifically in the R2-Ig domain, block pore function? Presumably reduction relieves conformational constraints on R2-Ig that prevent unfolding. Concurrent unfolding of the multiple copies of R2-VWA-Ig receptor bound to prepore might allow any of several possible inhibitory interactions. Because PA contains no Cys residues, there is no possibility of anomalous disulfides being formed between PA and reduced R2-VWA-Ig. However, noncovalent interactions between reduced receptor and PA, either during or following the conformational transition from the prepore to the pore, could interfere with formation of functional pore or its insertion into a membrane. Alternatively, interactions among the several reduced receptors bound to the pore, including intermolecular disulfide formation and/or noncovalent interactions, could generate a physical barrier to insertion of the pore into the membrane. It seems unlikely that the pore would form abortively due to an increase in the distance between the prepore and the membrane allowed by receptor unfolding, in view of the inhibitory effect on K^+^ release from liposomes, where the assay involves conformational transition of the R2-VWA-Ig:prepore complexes in solution.

Current concepts of the redox potentials within intracellular compartments support the notion that the endosomal lumen could be either oxidizing or reducing, depending on various endocytic pathways [33], [34]. It is believed that anthrax toxin-ANTXR2 complex traffics to late endosomes, where low-pH induces conformational transition of PA prepore-to-pore and translocation of LF/EF to the cytosol [35], [36], but which endocytic pathway ANTXR2 mediates the trafficking and the roles of cellular redox regulators in toxin action are not clear. The present study provides a novel venue to investigate the mechanism of anthrax toxin action and suggests novel strategies for anthrax toxin inhibition.
